# Recanalization Therapies in Acute Ischemic Stroke: Pharmacological Agents, Devices, and Combinations

**DOI:** 10.4061/2010/672064

**Published:** 2009-12-09

**Authors:** Vijay K. Sharma, Hock Luen Teoh, Lily Y. H. Wong, Jie Su, Benjamin K. C. Ong, Bernard P. L. Chan

**Affiliations:** Division of Neurology, Department of Medicine, National University Hospital, Singapore 119074

## Abstract

The primary aim of thrombolysis in acute ischemic stroke is recanalization of an occluded intracranial artery. Recanalization is an important predictor of stroke outcome as timely restoration of regional cerebral perfusion helps salvage threatened ischemic tissue. At present, intravenously administered tissue plasminogen activator (IV-TPA) remains the only FDA-approved therapeutic agent for the treatment of ischemic stroke within 3 hours of symptom onset. Recent studies have demonstrated safety as well as efficacy of IV-TPA even in an extended therapeutic window. However, the short therapeutic window, low rates of recanalization, and only modest benefits with IV-TPA have prompted a quest for alternative approaches to restore blood flow in an occluded artery in acute ischemic stroke. Although intra-arterial delivery of the thrombolytic agent seems effective, various logistic constraints limit its routine use and as yet no lytic agent have not received full regulatory approval for intra-arterial therapy. Mechanical devices and approaches can achieve higher rates of recanalization but their safety and efficacy still need to be established in larger clinical trials. The field of acute revascularization is rapidly evolving, and various combinations of pharmacologic agents, mechanical devices, and novel microbubble/ultrasound technologies are being tested in multiple clinical trials.

## 1. Introduction

Acute ischemic stroke (AIS) results in focal neurological deficits referable to a particular cerebral arterial territory. Most patients presenting with an AIS have arterial thrombi that occlude extracranial and/or intracranial arteries. Intravenously administered tissue plasminogen activator (IV-TPA) induces thrombolysis and remains the only FDA-approved thrombolytic agent for AIS within 3 hours from symptom-onset [1]. Fast dissolution of the thrombi and arterial recanalization in acute stroke often lead to dramatic clinical recovery [2].

The last decade has been a period of rapid advances in the management of AIS patients. Treatment in the acute phase is largely aimed at restoring blood flow in the affected artery, partially or completely. Since “time is brain”, timely recanalization of the occluded cerebral artery might result in restoration of cerebral perfusion, salvaging threatened ischemic tissue and improvement clinical outcome [3]. Therefore, success of recanalization therapy depends on timing as well as the degree of reperfusion. However, as with myocardial infarction, recanalization and reperfusion of ischemic tissue may occasionally exacerbate tissue damage by reperfusion injury, cerebral oedema, and hemorrhagic transformation [3].

Various pharmaceutical agents and devices, used alone as well as in combination, have been used to achieve recanalization in AIS. We aimed to review these approaches and the current understanding about recanalization in AIS.

## 2. Pharmacologic Agents

### 2.1. Intravenous Thrombolysis with TPA within Three Hours of Symptom Onset

Intravenous (IV) thrombolysis is effective when used within 3 hours of symptom onset in AIS, and tissue plasminogen activator (TPA) remains the only approved agent for this indication [1]. Although IV-TPA significantly improves patient outcomes, more than 50% of AIS patients still remain dependent or die. The strict time window and limited benefits have led to the evaluation of various alternative therapeutic strategies.

There have been multiple trials with IV-TPA in AIS on North America and Europe. The National Institute of Neurological Disorders and Stroke (NINDS) trials (part 1 and 2) [1] included patients treated within 3 hours of their symptom onset. European Cooperative Acute Stroke Study (ECASS 1 and 2) [4, 5] recruited patients up to 6 hours. Alteplase ThromboLysis for Acute Noninterventional Therapy in Ischemic Stroke (ATLANTIS) trial [6, 7] included North American patients (part A-up to 6-hours and part B in a 3–5-hour time window). A pooled analysis of these 6 large trials revealed that greater benefit is achieved if treatment is initiated within 90 minutes of symptom onset [Odds ratio (OR) 2.8, 95% Confidence interval (CI) 1.8–4.5], but potential benefit exists even beyond 3 hours (OR 1.4, 95% CI 1.05–1.85 for 180–270 minutes) [8]. This finding formed the basis of various studies for evaluating the extended “therapeutic window” in AIS.

### 2.2. Intravenous Thrombolysis with TPA beyond Three Hours

The Diffusion-weighted imaging Evaluation for Understanding Stroke Evolution (DEFUSE) [9] trial evaluated 74 patients presenting in the 3–6-hour time window with magnetic resonance imaging (MRI) to identify imaging patterns that might predict a favourable clinical response to TPA. The investigators found that a “target-mismatch” pattern (tissue at risk greater than 120% of tissue already damaged) on baseline MRI identified patients who benefited substantially from early reperfusion, while a “malignant” pattern (large portion of tissue already damaged) predicted severe intracranial hemorrhage following reperfusion. A “benign” pattern defined as small lesion on diffusion-weighted image (DWI), and perfusion-weighted imaging (PWI) was associated with a favorable clinical response regardless of subsequent reperfusion.

The Imaging Thrombolysis Evaluation Trial (EPITHET) [10] evaluated whether IV-TPA given 3–6 hours after stroke onset promotes reperfusion and attenuates infarct growth in patients with a mismatch in PWI-DWI MRI. Patients presenting within 3–6 hour time window underwent a baseline DWI-PWI MRI and then randomized to IV-TPA or placebo. MRI was repeated in 3–5 days and at 90 days to determine whether PWI-DWI mismatch can be used to select patients who derive benefit from IV-TPA, and whether a large DWI lesion predicts hemorrhagic transformation. Out of 101 patients recruited in this trial, 52 received IV-TPA compared to 49 patients in the placebo group. Mismatch on PWI-DWI MRI was seen in 85 of 99 (86%) patients. IV-TPA significantly improved reperfusion (and clinical outcomes) in patients who had mismatch. However; IV-TPA did not result in lower infarct growth (*P* = .054) [10].

A recent double-blind randomized placebo-controlled trial (European Cooperative Acute Stroke Study-ECASS III) [11] on 821 patients from 19 European countries, evaluated AIS treated with IV-TPA within 3–4.5 hours. Treated at a median time of 3 hours 59 minutes, patients receiving IV-TPA had favourable outcomes compared to placebo (52.4% versus 45.2%; OR 1.34; 95% CI 1.02–1.76; *P* = .04). Although the incidence of symptomatic intracranial hemorrhage (SICH) was higher with TPA (2.4% versus 0.2%; *P* = .008), mortality did not differ significantly between the two groups (7.7% and 8.4%, resp.; *P* = .68) [11]. The ongoing International Stroke Trial III (IST-III) [12], aimed at evaluating 3100 patients, is evaluating the safety and efficacy of IV-TPA within 6 hours of stroke onset.

Although arterial recanalization is one of the primary aims of thrombolysis, this phenomenon was not evaluated in major IV-thrombolysis trials [1, 4, 5, 7, 11]. Overall recanalization rates have varied from 30% to 92% during first 6–24 hours [13]. Higher recanalization ratea were noted during first hour (45%), followed by 11% during the second hour and only 7% additional patients achieved recanalization during next 4 hours [14]. Recanalization rates are determined by the site of occlusion also, with lower rates observed in proximal than in distal occlusions, probably due to higher clot burden. While almost one-third of middle cerebral artery (MCA) occlusions recanalize with IV-TPA, this occurs only in about 10% of patients with distal internal carotid artery (ICA) occlusions [15]. Furthermore, some associated conditions like coexisting carotid artery disease and diabetes mellitus may reduce the rates of recanalization [13]. Rha and Saver, in a recent meta-analysis found that the overall recanalization rate with any intervention was 55% [3].

## 3. Thrombolysis with Other Fibrinolytic Agents

### 3.1. Desmoteplase

Desmoteplase is a fibrin-specific and nonneurotoxic protein derived from the saliva of vampire bat. The Desmoteplase in Acute Ischemic Stroke (DIAS-1) trial was a dose-finding randomized, double-blind, placebo controlled, multicenter, phase II trial designed to evaluate the safety and efficacy of IV-Desmoteplase given in the 3–9 hour window [16]. In addition to the recanalization, DIAS trial evaluated reperfusion as an important outcome measure using the PWI-DWI mismatch. In initial phase of the trial, patients were randomized to fixed doses of Desmoteplase (25 mg, 37.5 mg, or 50 mg) or placebo. However, this was stopped early due to higher rates of SICH. In part II, weight-adjusted doses (62.5 *μ*g/kg, 90 *μ*g/kg, and 125 *μ*g/kg) were investigated. Reperfusion was assessed 4 to 8 hours posttreatment and defined as either ≥30% reduction of mean transit time volume or ≥2 point improvement in recanalization. Early reperfusion correlated favourably with clinical outcome (52.5% versus 24.6%; *P* < .002) and SICH occurred in 2.2%.

Efficacy and safety of IV desmoteplase was further evaluated in the Dose Escalation of Desmoteplase in Acute Stroke (DEDAS) study [17], in patients treated within 3 to 9 hour time window. Eligibility criteria included baseline NIHSS scores of 4–20 and MRI evidence of PWI-DWI mismatch. Results of DEDAS were almost similar to the DIAS trial. At a dose of 125 *μ*g/kg, Desmoteplase appeared to improve clinical outcome, especially in patients fulfilling the MRI criteria. Encouraged by these results, DIAS-2, a phase III study, was initiated with an additional attempt to investigate the clinical efficacy and safety of Desmoteplase in patients with “tissue-at-risk”, as assessed by PWI-DWI MRI or perfusion CT [18]. In this randomized, placebo-controlled, double-blind, dose-ranging study, patients “tissue-at-risk” were randomly assigned to 90 microgm/kg Desmoteplase, 125 microgm/kg Desmoteplase, or placebo within 3–9 hours after symptom onset. The primary endpoint was clinical response rate at day 90. Secondary endpoints included change in lesion volume between baseline and day 30, rates of SICH and mortality. Median baseline NIHSS score was 9 points and only 30% of the patients had a visible arterial occlusion at presentation. DIAS-2 study failed to show the benefit of Desmoteplase given 3–9 hours after the stroke onset using the MRI mismatch model.

## 4. Ancrod

Ancrod (Viprinex) is an enzyme derived from pit viper venom with defibrinating properties. The Stroke Treatment with Ancrod Trial (STAT) [19], a randomized, parallel-group, double-blind, placebo-controlled trial recruited patients from 48 centers in the United States and Canada. A total of 500 patients were randomly assigned to receive Ancrod (*n* = 248) or placebo (*n* = 252) as continuous 72-hour IV-infusion beginning within 3 hours of stroke onset, followed by infusions lasting 96 and 120 hours. The Ancrod regimen was designed to decrease plasma fibrinogen levels from 1.18 to 2.03 micromol/L. Favorable outcome was achieved by more patients in the Ancrod group (42.2% versus 34.4% with placebo; *P* = .04) with a trend toward more SICH in the Ancrod group (5.2% versus 2.0%; *P* = .06). However, phase-III trials with Ancrod (ASP I and II: Ancrod Stroke Program) used within 6 hours of symptom onset had to be terminated prematurely due to safety concerns [12]. Levy et al. suggested that some modifications to Ancrod dosing may substantially improve efficacy while reducing SICH [20].

## 5. Other Agents

Although not thrombolytic, heparin and glycoprotein IIb/IIIa receptor antagonists have been investigated in AIS. In a study of 312 patients treated with low molecular weight heparin (Nadroparin) within 48 hours of symptom onset, the investigators evaluated 2 doses of Nadroparin against placebo and observed a significant dose-dependent effect in favor of Nadroparin (*P* = .005). However, no significant differences were observed in secondary outcomes at 10 days [21]. The Heparin in Acute Embolic Stroke Trial (HAEST) compared Dalteparin against aspirin in atrial fibrillation [22]. Patients were recruited within 30 hours of symptom onset. The frequency of recurrent AIS during the first 14 days was 19/244 (8.5%) in Dalteparin-allocated patients versus 17/225 (7.5%) with aspirin (OR1.13, 95% CI 0.57–2.24). The secondary events (SICH, stroke progression and death) during the first 14 days also revealed no additional benefit of Dalteparin. There were no significant differences in functional outcome or death [22]. 

Abciximab, a chimeric mouse/human monoclonal antibody with high affinity for platelet glycoprotein IIb/IIIa receptor, has been used as an adjunct to endovascular procedures or a thrombolysis in animal model [23] and for treatment of patients with AIS [24]. Furthermore, a dose-escalation study in 74 subjects suggested low risks of SICH when it was given up to 24 hours after stroke onset [25]. Similar safety and efficacy were noted in a larger randomized trial on 400 AIS subjects treated within 6 hours [26]. Despite this, the Abciximab in Emergency Treatment of Stroke Trial Abciximab (AbESTT-II), a phase III trial was terminated prematurely due to an unfavourable benefit-risk profile [27]. Within 5 days of enrolment, 5.5% of Abciximab-treated (compared to 0.5% on placebo) patients developed SICH (*P* = .002). The trial did not demonstrate any improvement in outcomes with Abciximab.

The Reopro Retevase Reperfusion of Stroke Safety Study (ROSIE) is an ongoing open-label, dose escalation, safety and proof-of-principle study [12]. It is including AIS patients within 3–24 hours of symptom onset, with a disabling neurological deficit, NIHSS score ≤16 points, and evidence of a perfusion deficit on PWI MRI and MRA. This trial is looking into the safety and efficacy of Abciximab (ReoPro) and Reteplase (Retevase) in reperfusing the ischemic tissue. ROSIE-CT is similar to ROSIE and includes patients ineligible for MRI, and CT perfusion would assess reperfusion [12]. Both these trials have completed recruitment and the results are awaited.

### 5.1. Intra-Arterial Thrombolytics

Intra-arterial (IA) thrombolysis offers potential advantage of higher concentrations of thrombolytic delivered to the clot with reduced systemic exposure and the possibility of higher rates of recanalization. While there are currently no FDA-approved IA thrombolytic agents, several uncontrolled and anecdotal studies have evaluated IA thrombolysis in AIS.

In an open clinical series, IA thrombolysis has shown higher early recanalization rates (50% to 80%) than IV therapy (30% to 50%) [15]. The Prolyse in Acute Cerebral Thromboembolism (PROACT-I) trial was a randomized, double-blinded, placebo-controlled phase II study evaluating the recanalization safety and efficacy of IA recombinant prourokinase (r-proUK) in patients with symptomatic M1 or M2 MCA occlusions at 2 hours after initiation of treatment [28]. Of 1314 patients screened, 40 (3%) were randomized to r-proUK (6 mg) or placebo infused IA over 2 hours. At the end of infusion, significantly higher recanalization rates were observed in the treatment group (57% versus 14.3%; *P* = .017). To determine the phase III clinical safety and efficacy of r-proUK, PROACT II was conducted [29]. Patients presenting within 6 hours of AIS were randomized to either 9 mg of IA r-proUK plus heparin or heparin alone, over 2 hours. Of 12323 patients screened, 180 patients were randomized. With 5.3 hours as the median time to initiate therapy, 66% of the patients in the treatment arm achieved MCA recanalization compared with 18% in “heparin only” arm (*P* < .001). Likewise, 40% of the patients in the treatment group achieved functional-independence at 90 days compared with 25% in “heparin only” arm (OR 2.13, 95% CI 1.02–4.42; *P* = .04). Despite higher rates of ICH in treatment group (10% versus 2%; *P* = .06), no differences were noted in mortality between the two groups [29]. Based on a reanalysis of the PROACT II trial, patient selection with a noncontrast CT scoring could identify patients who benefit from IA thrombolysis once the 3-hour time window for intravenous thrombolysis has passed [30].

## 6. Combined Intravenous-Intra-Arterial Thrombolysis

IV thrombolysis is the fastest way to initiate treatment. Conversely, IA thrombolysis requires an experienced interventionalist, angiographic facilities, and time to deploy the endovascular in the culprit vessel. This imposes treatment initiation delays. To achieve better rates of recanalization and clinical outcomes, a combination of these two modalities in AIS have been evaluated. The first pilot study was the Emergency Management of Stroke (EMS) trial, a randomized, placebo-controlled multicenter study designed to test the feasibility, relative benefits, and risks of combined IV/IA thrombolysis [31]. Within 3 hours of stroke onset, the IV/IA treatment group (*n* = 17) received 0.6 mg/kg of IV-TPA (10% bolus, 60 mg maximum over 30 minutes) followed within 2 hours by local IA-TPA (maximum 20 mg). The control group (*n* = 18) received IV placebo followed similarly by IA-TPA. Although the treatment group showed higher rates of angiographic recanalization than controls (54% versus 10%; *P* = .03) and lower NIHSS scores at 90 days, significantly higher mortality was noted in the treatment group (29% versus 5.5%; *P* = .06).

To further investigate the combined approach, the Interventional Management of Stroke (IMS-I) study was performed [32]. This multicenter open-label trial had the design similar to EMS and aimed at testing the feasibility and safety of combined IV/IA-TPA within 3 hours of symptom onset by comparing outcomes to matched (age and baseline NIHSS) historical controls from the NINDS trial [1]. The combination therapy was found to be feasible with safety similar to IV-TPA alone. Outcomes in the IMS subjects were also significantly better at 3 months than the placebo subjects of NINDS trial [32]. Encouragement from these findings led to the ongoing IMS-III, which is a randomized, multicenter, open-label clinical trial of 900 subjects assessing whether the combined IV/IA approach is superior to standard IV-therapy alone when initiated within 3 hours of symptom onset [12].

Pending further short-and long-term data on the benefit of combined IV/IA therapy compared with IV therapy alone, current guidelines suggest that IV-therapy should not be withheld from eligible patients. IA-thrombolysis is a therapeutic option for selected patients who have major stroke of <6 hours duration due to occlusions of the MCA and not suitable for IV-TPA. Treatment requires the patient to be at an experienced stroke center with immediate access to cerebral angiography and qualified interventionalists [32].

## 7. Devices for Treatment of Acute Ischemic Stroke

Pharmacologic agents have risks and contraindications that have prompted the development of devices as alternative ways to restore blood flow. In addition, the efficacy of pharmacologic agents can be augmented by a variety of devices. The Merci Retrieval System (Concentric Medical, Inc.) is an FDA-approved device comprised of a tapered nitinol wire with helical loops of decreasing diameter at its distal end. It is advanced through a microcatheter directly through the clot and then released to acquire the helical loop shape and retrieve the clot.

To evaluate safety and efficacy of Merci device, the Mechanical Embolus Removal in Cerebral Ischemia (MERCI) single arm, multicenter trial evaluated 141 patients presenting within 3–8 hours of symptom-onset [33]. With a mean procedural time of 2.1 hours, 48% of patients had successful recanalization of MCA, terminal ICA, vertebral or basilar artery. This was in comparison to 18% recanalization in the PROACT-II historical control arm (*P* < .0001) [29]. Adjuvant therapy (IA-TPA with or without angioplasty/snare) was needed in 51 of the 141 eligible patients. Clinically significant procedural complications and ICH were seen in 7.1% and 7.8%, respectively. More patients with successful recanalization achieved good neurological outcomes at 90 days than those with unsuccessful recanalization (46% versus 10%; *P* < .0001) [34]. 

FDA approval of the MERCI Retriever as a device for clot removal in AIS engendered criticism from the stroke neurologists who cited double standards between drug and device regulatory requirements for approval [35]. Despite the FDA approval, the clinical utility of the MERCI device has not been established.

The ongoing Magnetic Resonance and Recanalization of Stroke Clots Using Embolectomy (MR-Rescue) trial uses MRI to compare embolectomy with the Merci devices to standard treatment in AIS patients presenting within 8 hours of symptom onset [12]. Mechanical devices offer some distinct advantages over pharmacological thrombolysis like faster recanalization [15], effectiveness in removing large thrombi, and lower risk of SICH. However, these devices require bulky catheters and may cause endothelial damage, vessel wall perforation, and intracranial bleeding. Recanalization is believed to be the key for better outcomes in AIS. However, it has not been evaluated routinely in the clinical trials. Comparative rates of recanalization and methodology of some of the major trials in acute stroke are summarized in the Table 1.

## 8. Combining Pharmacology and Devices

Simple mechanical clot disruption with microcatheter or microwire, or aggressive mechanical clot disruption (AMCD) with a snare or balloon angioplasty, in conjunction with thrombolysis, may achieve higher rates of recanalization. Quershi et al. treated 19 patients with balloon angioplasty and/or snare in addition to IV/IA thrombolysis, for both proximal and distal occlusions [36]. Although recanalization rate was 86%, the mortality rate was 53%. Noser et al. employed AMCD by microcatheter/microwire clot maceration, angioplasty, stent, or snare in 32 patients (adjuvant to IA and/or IV thrombolysis) [37]. Seventy-five percent of patients showed recanalization with favorable outcome in 59%. The mortality rate was 12.5%.

Endovascular therapy (percutaneous transluminal angioplasty with or without stent placement) in combination with IV/IA thrombolysis +/− glycoprotein IIb/IIIa inhibition has been used for MCA as well as extracranial ICA occlusions with varying results in several small case series[38–40].

Multi-MERCI was an international, multicenter, prospective, single-arm trial of thrombectomy in patients with large vessel stroke treated within 8 hours of symptom-onset [41]. It allowed inclusion of patients with persistent large vessel occlusion after IV-TPA and permitted use of adjuvant IA thrombolysis, if the device failed. One hundred sixty-four patients with a median NIHSS of 19 points underwent mechanical thrombectomy. Successful recanalization was achieved in 57.3% of treatable vessels and improved to 69.5% when mechanical embolectomy was combined with IA-TPA. The trial concluded that favourable clinical outcomes (36% of patients) were significantly related to arterial recanalization. Clinically significant procedural complications were observed in 5.5% of patients [41].

Ultrasound exposure has been shown to help in dissolving the thrombus [42]. Experimental evidence suggests that thrombolytic effect of IV-TPA is substantially enhanced by ultrasound, particularly if used in low MHz-kHz frequency range. Ultrasound exposure causes reversible disaggregation of uncrosslinked fibrin fibers and microcavity formation in the shallow layers of thrombus, allowing increased penetration of TPA into the clot and enhancing flow with microstreaming and vessel dilation [43–45].

Lytic activity of IV-TPA can be safely augmented with TCD. The Transcranial low-frequency Ultrasound Mediated Thrombolysis in Brain Ischemia (TRUMBI) trial, in a phase II randomized, multicenter study tested the efficacy of low-frequency ultrasound exposure along with IV-TPA in AIS [46]. An experimental, nonimaging therapeutic device operating with approximately 300 kHz pulsed wave ultrasound was used. The study was terminated after enrolling only 14 patients in the treatment arm due to a significantly high incidence of SICH. These harmful bioeffects of low-frequency ultrasound were probably due to lack of prior human dose-escalation studies. Using ultrasound in the mega-Hertz range with transcranial duplex equipments was recently tested by Eggers et al. [47]. Patients with acute occlusions of MCA were randomized into a target group receiving 1-hour transcranial continuous insonation using a 1.8-MHz Doppler ultrasound probe or a control group during standard thrombolysis with IV-TPA. Patients in the ultrasound group showed greater neurological improvement. However, 15.8% of patients developed SICH as compared to 5.6% in the controls (*P* = .60).

Combined Lysis of Thrombus in Brain Ischemia Using Transcranial Ultrasound and Systemic tPA (CLOTBUST) was a phase II randomized, double-blind, placebo-controlled trial evaluating the feasibility and safety of IV-TPA and continuous 2-hour monitoring with commercially available FDA approved 2 MHz diagnostic TCD devices [48]. Complete recanalization or dramatic clinical recovery within 2 hours after administration of IV-TPA occurred in 49% of those receiving TCD compared with 30% receiving placebo (*P* = .03). Recently, we reported the feasibility, safety, and efficacy of ultrasound-assisted thrombolysis in Singaporean patients [49].

In the pivotal CLOTBUST trial, primary endpoint was achieved in 49% of patients in the target-group and 30% in the controls (*P* = .03) [48]. CLOTBUST was not powered to evaluate the efficacy of ultrasound-enhanced thrombolysis in improving functional outcome. A trend toward better functional outcomes in patients treated with continuous TCD monitoring merits a pivotal phase III clinical trial. However, larger number of patient needed to power such a clinical trial coupled with small number of neurosonologists and emergence of newer therapies posed serious limitations. Our multicenter collaborative group prospectively implemented a protocol for TCD assessment of intracranial recanalization with IV-TPA [50]. We aim to determine whether early recanalization is independently associated with better 3-month outcome in AIS patients with intracranial arterial occlusions. Additionally, it would seek to recruit and train centres and operators before embarking into a large randomized phase III clinical trial.

The IMS-II trial evaluated IV versus IV/IA TPA along with the EKOS MicroLysus catheter (Bothell, WA), which directly exposes the clot to low power ultrasound [51]. The recanalization rate of grade 2 and 3 in EKOS-treated subjects in IMS II was 45.5% as compared with 30% in IMS I-treated subjects (*P* = .26) [51].

One novel method of enhancing the rates of recanalization by IV-TPA could be the use of gaseous microspheres (*μ*S) during TCD monitoring. Gaseous *μ*S are used as contrast agents for ultrasound imaging and their exposure to pulsed TCD leads to their compression, oscillation, and collapse. These physical alterations could transmit mechanical energy momentum to surrounding fluids, accelerate the residual flow, and possibly help in disrupting the thrombus [52, 53]. Recently, promising human studies were done with TCD-activated first generation gaseous *μ*S as an adjunctive therapy with IV-TPA [54, 55]. The Safety Study of Microbubbles With Transcranial Ultrasound and TPA in the Management of Acute Ischemic Stroke (TUCSON) trial*, w*as initiated with 3rd generation submicron-sized microspheres [56]. This was a phase I/II, randomized, placebo-controlled, single-blind, dose escalation, safety/efficacy study, however, the study had to be terminated prematurely due to an increased SICH in the 2nd cohort [12].

Various other innovative approaches have been adopted to achieve better clinical outcomes in AIS. The NeuroThera Effectiveness and Safety Trial–2 (NEST-2) tested the safety and efficacy of transcranial laser therapy (TLT) [57]. This double-blind, randomized study compared TLT treatment to sham control included 660 patients (331 received TLT and 327 received sham) within 24 hours of symptom onset. More patients in the TLT group achieved favorable outcome versus the sham group (*P* = .094) but no prespecified test achieved significance. A post-hoc analysis of patients with a baseline NIHSS score of <16 showed a favourable outcome at 90 days on the primary endpoint (*P* < .044). Mortality rates and serious adverse events did not differ between groups. A definitive trial with refined baseline NIHSS criteria is being planned.

## 9. Conclusions

Acute is a chemic stroke which continues to be a devastating disease. Early access to definitive therapy remains the key for a better outcome. The search for newer, more effective and yet, safer pharmacological, interventional, and combined treatments continues. The diversity of new emerging approaches makes stroke an exciting and rapidly evolving field.

## Figures and Tables

**Table 1:** Arterial occlusions and recanalization patterns in some clinical trials in acute ischemic stroke.

TRIAL			Treatment	Number of patients	Highest reported recanalization rate (%)	Assessment of recanalization
NINDS	Active		IV-TPA	312	—	None
	Control		Placebo	312	—	

DIAS	Active		Desmopletase (IV)	71 (Total)	49	MRA-complete or partial recanalization
	Part-l	25 mg		16	56	
		37.5/50 mg		13	46	
	Part-2	62.5 mg/kg		13	23
		90 mg/kg		15	47
		125 mg/kg		14	71
	Control		Placebo	26 (Total)	19
	Part-1			16	19
	Part-2			10	20

PROACT-II (TIMI 2 + 3)	Active		lAr-proUK	121	66	Cerebral angiography (TIMI 2 + 3)
	Control		Placebo	59	18	

EMS	Active		IV + IA TPA	11	81	Cerebral angiography
	Control		Placebo + IA TPA	10	50	

MERCI	Active		MERCI ± Lytic			Cerebral angiography (any recanalization)
	MCA			80	45	
	ICA			47	53	
	VAIBA		—	14	50	
	Control			—	—	

CLOTBUST (at 2 hours)	Active		IV-TPA + TCD	63	38	TCD
	Control		IV-TPA	63	13	

TRUMBI	Active		IV-TPA + Ultrasound	14	29.3	MRA
	Control		IV-TPA	12	50.0	
